# Correction to: Esophageal cancer practice guidelines 2017 edited by the Japan Esophageal Society: part 1 and Part 2

**DOI:** 10.1007/s10388-022-00935-4

**Published:** 2022-06-27

**Authors:** Yuko Kitagawa, Takashi Uno, Tsuneo Oyama, Ken Kato, Hiroyuki Kato, Hirofumi Kawakubo, Osamu Kawamura, Motoyasu Kusano, Hiroyuki Kuwano, Hiroya Takeuchi, Yasushi Toh, Yuichiro Doki, Yoshio Naomoto, Kenji Nemoto, Eisuke Booka, Hisahiro Matsubara, Tatsuya Miyazaki, Manabu Muto, Akio Yanagisawa, Masahiro Yoshida

**Affiliations:** 1 Department of Surgery, Keio University School of Medicine, 35 Shinanomachi, Shinjuku‑ku, Tokyo, 160‑8582 Japan; 2 Department of Radiology, Graduate School of Medicine, Chiba University, Chiba, Japan; 3 Department of Gastroenterology, Saku Central Hospital, Nagano, Japan; 4 Gastrointestinal Medical Oncology Division, National Cancer Center Hospital, Tokyo, Japan; 5 Department of Gastrointestinal Tract Surgery, Fukushima Medical University School of Medicine, Fukushima, Japan; 6 Department of Endoscopy and Endoscopic Surgery, Gunma University Hospital, Maebashi, Gunma Japan; 7 Department of General Surgical Science, Gunma University Graduate School of Medicine, Maebashi, Gunma Japan; 8 Department of Surgery, Hamamatsu University School of Medicine, Hamamatsu, Shizuoka Japan; 9 Department of Gastroenterological Surgery, National Kyushu Cancer Center, Fukuoka, Japan; 10 Department of Gastroenterological Surgery, Osaka University Graduate School of Medicine, Suita, Osaka Japan; 11 Department of General Surgery, Kawasaki Medical School, Okayama, Japan; 12 Department of Radiation Oncology, Yamagata University School of Medicine, Yonezawa, Japan; 13 Department of Frontier Surgery, Graduate School of Medicine, Chiba University, Chiba, Japan; 14 Department of Clinical Oncology, Kyoto University Hospital, Kyoto, Japan; 15 Department of Pathology, Kyoto Prefectural University of Medicine, Kyoto, Japan; 16 Department of Hemodialysis and Surgery, Chemotherapy Research Institute, International University of Health and Welfare, Ichikawa, Japan

## Correction to: Esophagus (2019) 16:1–24 https://doi.org/10.1007/s10388-018-0641-9 and Esophagus (2019) 16:25–43 https://doi.org/10.1007/s10388-018-0642-8

In the published guidelines, the Conflict of interest section should be as follows.

Funds to support the development of these guidelines were provided by the Japan Esophageal Society. Members of the Guideline Review Committee and Guideline Steering Committee personally reported their conflicts of interests in conformity with the regulations of the Japan Esophageal Society. The Ethics Committee and the Board of Directors of the Japan Esophageal Society confirmed the personally reported conflict-of-interest situations. Situations of conflicts of interests are uploaded on the website of the Japan Esophageal Society for each fiscal year.

